# Dielectric Spectroscopy Based Detection of Specific and Nonspecific Cellular Mechanisms

**DOI:** 10.3390/s21093177

**Published:** 2021-05-03

**Authors:** Michael R. Stoneman, Valerică Raicu

**Affiliations:** 1 Department of Physics, University of Wisconsin-Milwaukee, Milwaukee, WI 53211, USA; stonema2@uwm.edu; 2 Department of Biological Sciences, University of Wisconsin-Milwaukee, Milwaukee, WI 53211, USA

**Keywords:** dielectric spectroscopy, dielectric relaxation, G protein-coupled receptor, GPCR, ligand binding, label-free detection

## Abstract

Using radiofrequency dielectric spectroscopy, we have investigated the impact of the interaction between a G protein-coupled receptor (GPCR), the sterile2 α-factor receptor protein (Ste2), and its cognate agonist ligand, the α-factor pheromone, on the dielectric properties of the plasma membrane in living yeast cells (*Saccharomyces cerevisiae*). The dielectric properties of a cell suspension containing a saturating concentration of α-factor were measured over the frequency range 40Hz–110 MHz and compared to the behavior of a similarly prepared suspension of cells in the absence of α-factor. A spherical three-shell model was used to determine the electrical phase parameters for the yeast cells in both types of suspensions. The relative permittivity of the plasma membrane showed a significant increase after exposure to α-factor (by 0.06 ± 0.05). The equivalent experiment performed on yeast cells lacking the ability to express Ste2 showed no change in plasma membrane permittivity. Interestingly, a large change also occurred to the electrical properties of the cellular interior after the addition of α-factor to the cell suspending medium, whether or not the cells were expressing Ste2. We present a number of different complementary experiments performed on the yeast to support these dielectric data and interpret the results in terms of specific cellular reactions to the presence of α-factor.

## 1. Introduction

G protein-coupled receptors (GPCRs), the largest family of cell-surface receptors, regulate a broad array of cellular functions, making them of the utmost physiological importance [1,2]. GPCRs initiate the signal transduction processes used by eukaryotic cells to facilitate intercellular communication and sense environmental stimuli. While the triggering mechanisms for GPCRs are diverse, a significant percentage of these receptors are activated through the binding of a peptide to their extracellular domain [3]. Upon ligand binding, the receptor undergoes a slight conformational change, setting off a cascade of intracellular events, which eventually lead to a change in cellular behavior. Methods that can quantify the degree of interaction between ligands and the GPCRs to which they potentially bind are of great importance, and development of these methods remain a major focus of the drug-discovery research field.

There exists a large number of in vitro functional assays that have been successfully utilized to determine the effectiveness of a ligand in activating a specific signaling cascade [4,5]. These functional assays are typically based on detecting changes in intracellular levels of a labelled secondary messenger (such as calcium ions or cyclic AMP), which occur as a result of a specific ligand-induced signaling cascade and therefore typically require knowledge regarding the specific G-protein activation pathway (Gαs, Gαi/o, or Gαq) initiated by binding of the ligand. However, for several GPCRs, the receptor­mediated signaling pathways are unknown, thus excluding the use of many of these cell-based assays. Furthermore, different agonists can cause the same receptor to preferentially activate different downstream pathways (known as biased agonism), which necessitates the use of more than one functional assay for determining whether or not the ligand is bound to the receptor.

Binding of a ligand to a GPCR is also routinely assessed by labelling the ligand of interest, either with a radioactive isotope or fluorescent marker [6,7,8]. The use of radio-labeled ligands in either kinetic, saturation, or competition binding assays provides a reliable way to determine binding affinities between said ligand and a particular receptor. However, due to issues involved with the safety, expense, and general handling of radioactive isotopes, efforts have been made in recent years to move away from the use of radioactive isotopes and replace them with fluorescent labels. The usefulness of fluorescently labelled ligands [9,10,11] can actually extend beyond a simple replacement of radioactive isotopes in binding assays and be used for other more in depth studies [7,8], such as localization of bound receptors using traditional fluorescence microscopes to more sophisticated approaches that quantify the geometry of ligand–receptor and receptor–receptor complexes using Forster resonance energy transfer [12]. However, attachment of a fluorescent probe to the ligand can potentially alter the nature of the ligand’s interaction with receptors. For example, multiple variants of the yeast peptide mating pheromone α-factor labelled with a small fluorophore (NBD) exhibited reduced binding affinity for the receptor, with the binding affinities of several of the variants even reduced to undetectable levels [13].

The techniques of Förster resonance energy transfer (FRET) [14,15,16] and bioluminescence resonance energy transfer (BRET) [17] have been crucial to the progression of knowledge regarding the activities of GPCRs. However, a high degree of uncertainty still clouds the use of these methods for detection of the agonist based GPCR activation. While some investigators have reported large changes to FRET- and BRET-derived signals upon agonist binding to certain GPCRs [17,18,19,20,21,22], others have failed to detect any difference in signal after agonist stimulation of other receptors [23,24,25,26,27]. One challenge that such methods face is that agonist-induced conformational changes to the receptors of interest are not necessarily accompanied by significant changes in the position and/or orientation of the fluorescent tags needed to detect the FRET or BRET signal [28]. Therefore, competent direct “label-free” methods to detect in vivo agonist and antagonist binding to GPCRs remain important.

The electrical properties of biological cells and tissues have long been of use to biological investigators, beginning with the observation that cells, when exposed to a direct current, stored some of the energy of the electric field, and released it later when the external current source was removed [29]. This pioneering discovery paved the way for the field of dielectric spectroscopy, a measurement technique that capitalizes on the frequency dependence of permittivity and conductivity (or dielectric dispersion) exhibited by biological cells subjected to an alternating electric field [30]. Dielectric spectroscopy has been utilized to discern the macroscopic electrical properties of a multitude of biological cell suspensions and cell tissue types [29,30,31,32,33,34]. Furthermore, it has also been highly effective in revealing information regarding time-dependent cellular movement—e.g., cell sedimentation [35], aggregation [36], division [37], and differentiation [38]. Recent advances in the field have demonstrated the ability to model more complex cellular architectures, such as those that present hierarchical relationships between their parts—i.e., fractals [39,40]. Finally, it has recently been shown that contactless and reusable sensors, which are capable of highly accurate dielectric characterization of liquids, can be manufactured in a low cost, easy-to-fabricate manner [41,42].

A significant advance in dielectric spectroscopy occurred when the theories developed by both Maxwell and Wagner to describe the behavior of inhomogeneous dielectrics in external electric fields [43,44] were applied to biological systems [45]. This pinpointed the mechanism of interfacial polarization, the accumulation of charge at the interfaces separating regions of differing electrical properties, as the chief contributor to the dielectric dispersion of biological cells in the radiofrequency range, typically referred to as the beta dispersion. With the help of such models, an analysis of the beta dispersion reveals information corresponding to the dielectric properties of various compartments of the heterogeneous cells under study—e.g., the low conducting membranes surrounding the cell and interior vesicles, as well as the cytosol. Due to this striking ability to noninvasively discern the electrical parameters of various cellular regions, the technique of multifrequency dielectric spectroscopy has been successfully employed to monitor the response of subcellular compartments in vivo while subjecting cells to various environmental changes expected to affect said cellular regions [46,47,48].

Multifrequency dielectric spectroscopy measurements of cell suspensions can pinpoint the regions within the cells that are responsible for the change in electrical properties and determine whether these changes are due to physiological processes occurring within the cell. Two essential tools are needed in order to effectively implement this type of analysis on dielectric data obtained. The first is an accurate electrical model describing the particular cells of interest. In this regard, the *saccharomyces cerevisiae* (yeast cell) is an excellent test system for dielectric spectroscopy studies [49,50]. It has been previously shown that the radiofrequency dielectric spectra of yeast cells may be simulated using the spherical three-shell model [50]. Biologically speaking, the choice of yeast cells for experimentation is also advantageous as yeast cells are easy to grow and manipulate. Furthermore, the yeast system is also advantageous for this particular study as yeast cells have only two types of GPCRs, a pheromone receptor and a glucose-sensing GPCR, the Gpr1. Therefore, the smaller variety of GPCRs in yeast cells (as compared, for example, to the hundreds of GPCRs found in human cells) lends itself as a good model to study GPCR activity. The second essential component in simulating the measured dielectric data of biological cells with morphological electrical models is a reliable algorithm that is able to not only simulate the obtained cell dielectric measurements, but also determine the uniqueness of the cell compartment dielectric properties gleaned from measurements. A proper model for the highly complex biological cell requires a large number of both morphological and electrical parameters associated with the various insulating and conducting compartments of the cells. Fortunately, there exists a well-established powerful global minimum search algorithm known most commonly as simulated annealing [51], which addresses these technical concerns in the data fitting process.

The purpose of this report is to demonstrate the ability of dielectric spectroscopy to discern electrical changes within subcellular compartments of biological cells following the binding of agonists to a specific GPCR expressed by yeast cells. We have performed dielectric spectroscopy measurements upon yeast cells expressing the physiologically relevant sterile2 α-factor receptor (Ste2) exposed to saturating concentrations of the natural binding ligand of Ste2, the α-factor. Ste2, a member of the GPCR family, is expressed in yeast cells of the **a** mating type and binds the pheromone, α-factor, secreted by yeast cells of the **α** mating type. The binding of α-factor to Ste2 initiates a signal transduction pathway that leads to cell division arrest and mating with an **α** type yeast cell [20,52,53]. Equipped with an applicable electrical model for yeast and a robust data fitting routine, we extracted the dielectric properties of not only the plasma membrane region where the Ste2 receptors are expected to bind the α-factor, but also of the interior of the cell. The permittivity of the plasma membrane showed a significant change when the cells were subjected to α-factor, demonstrating the ability of dielectric spectroscopy to sense the binding of ligand to receptors in the membrane. Remarkably, significant changes to the permittivity and conductivity of the electrolytic medium making up the cytoplasm also occurred when the cells were exposed to α-factor, indicating that dielectric spectroscopy possesses the sensitivity to detect internalization processes being carried out by the cell. The reported dielectric results are corroborated by the results of an array of complementary measurements performed on the yeast cells. 

## 2. Materials and Methods

### 2.1. Yeast Sample Preparation 

The strain of *saccharomyces cerevisiae* used in this study (KBY58; *MATa* *leu2-3,112 ura3-52 his3*-Δ*1 trp1 sst1*-Δ*5 ste2*Δ) lacked a functional copy of STE2 in the chromosome [54]. These cells were genetically engineered to express one of two different types of plasmid pairs. One type of cell sample contained a pair of plasmids,(pYF1988 and pYF2034), each of which contained a gene encoding the sterile2 α-factor receptor fused in frame to a gene encoding one of two variants of the green fluorescent protein (GFP)—i.e., either GFP2 [55] (PYF1988) or YFP [56] (PYF2034). Ste2 is the protein of interest in this study, while the GFP2 and YFP tags serve as markers of the Ste2 in separate fluorescence microscopy-based studies of this receptor [20,53,57]. The other type of cell sample, referred to as Ste2Δ throughout the manuscript, contained a pair of plasmids comprising the same DNA as the first plasmid pair except those coding DNA for the Ste2–fluorescent protein complex. Both types of cells were grown on synthetic solid medium plates lacking the nutrients uracil and tryptophan, which were needed to select for both plasmids. After three days of growth, yeast cell colonies were scraped from the solid medium, resuspended in deionized water, deposited on a fresh solid medium plate as a drop, and then the plate was tilted so the cell suspension formed a streak. These plates were incubated for 40 h at 30 °C. Cells were then scraped from the streak and suspended in 50 mL of synthetic liquid medium, also lacking uracil and tryptophan, and cultured at 30 °C for 48 h until the cells reached the early stationary phase (optical density = 2.3).

At this time, the liquid culture was centrifuged for two minutes at 1500× *g*, and the remaining pellet resuspended in 1 mL of 0.01 M KCl for 45 min in order to wash out excess electrolyte from the cellular interior. The cells were again separated by centrifugation and resuspended in 0.02 M KCl for 45 min. The cells were then centrifuged and resuspended for the final time in 0.02 M KCl, where they were allowed to equilibrate with the suspending medium for approximately 20 min. At this point, both types of cell suspension (Ste2Δ- and Ste2-expressing) were split into two equal volumes of 390 μL. To one of volumes, 10 µL of 10 mM α-factor in 100 mM sodium acetate at pH 5.2 solution was added (Zymo Research Corporation, Orange, CA, USA); the final concentration of the α-factor ligand in the cellular suspension was 250 µM. To replicate this treatment, save for the addition of the α-factor, 10 µL of 100 mM sodium acetate solution was added to the other volume of cells. To reiterate, this treatment of splitting the cell suspension into two equivalent volumes and adding α-factor in sodium acetate solution to one and the equivalent volume of only sodium acetate solution to the other was carried out for both Ste2Δ- and Ste2-expressing suspensions of cells. The cells were allowed to equilibrate with this slightly altered suspending medium for approximately 20 more minutes. After this equilibration time, dielectric measurements of all four types of cell samples (Ste2-expressing, Ste2-expressing exposed to α-factor, Ste2Δ, and Ste2Δ exposed to α-factor) were executed according to the protocol described in Section 2.2.1. In parallel to the dielectric measurements, wide field microscopy images were taken of both Ste2Δ- and Ste2-expressing cells in order to obtain detailed size measurements for each of the four variants of samples. 

A slight variation of the cell sample preparation was used to investigate whether the addition of molecules to the yeast cell exterior at the concentration used for α-factor (250 µM) had a significant impact upon the osmotic pressure sensed by the cells. In this regard, Ste2Δ yeast cells (i.e., yeast cells not expressing Ste2) were exposed to the sugar alcohol Sorbitol (Fisher Scientific, Pittsburgh, PA, USA) at the same molar concentrations as was used for α-factor molecules. The cells were grown, harvested and washed in the same exact manner as reported above, and the final suspension of cells was once again split into two equal volumes. A 10 µL solution of 10 mM Sorbitol in 100 mM sodium acetate was added to one of the volumes, while a 10 µL solution of 100 mM sodium acetate was added to the other. The cells were allowed to equilibrate with the slightly altered suspending medium, i.e., either Sorbitol in sodium acetate or just sodium acetate added to it, for 20 or more minutes. After this equilibration time, dielectric measurements of the cell suspensions were executed according to the protocol described in Section 2.2.1. 

A second variation to the yeast sample treatment was implemented for the purpose of obtaining fluorescence measurements of yeast cells exposed to D-Luciferin molecules (D-Luciferin Potassium Salt, GoldBio, St Louis, MO, USA). KBY58 cells were grown on standard YPD agar plates containing 1% Bacto™ Yeast Extract, 2% Bacto™ Peptone, and 2% D-glucose (Thermo Fisher Scientific Inc., Millersburg, PA, USA). Cells were scraped from the plate, suspended in YPD solution, and grown overnight. Four mL of cell was then centrifuged and resuspended in 100 μL of 100 mM KCl solution for 40 min. Two equal volumes (206 μL) were then separated from the cell suspension. One of the volumes was left centrifuged and resuspended again simply in 100 mM KCl. In other words, this sample was left untreated in order to obtain the emission spectrum and quantify the level of intrinsic fluorescence (or autofluorescence) inherent to the yeast cells. To the second volume, 10 μL of 5.15 mM D-Luciferin solution was added to achieve a D-Luciferin concentration of 250 μM. After 25 min of exposure to D-Luciferin, these cells were washed three times with 1 mL of 100 mM KCl, and finally resuspended in 50 μL of 100 mM KCl. Fluorescence images of random cells from both volumes were then obtained. Fluorescence measurements of yeast cells in the presence and absence of D-Luciferin were performed on cells lacking fluorescently labelled Ste2 in order to avoid contributions of the fluorescent markers in the Ste2-expressing cells to the measured D-Luciferin signal. 

### 2.2. Measurement Techniques

#### 2.2.1. Dielectric Measurements

**Figure 1:**
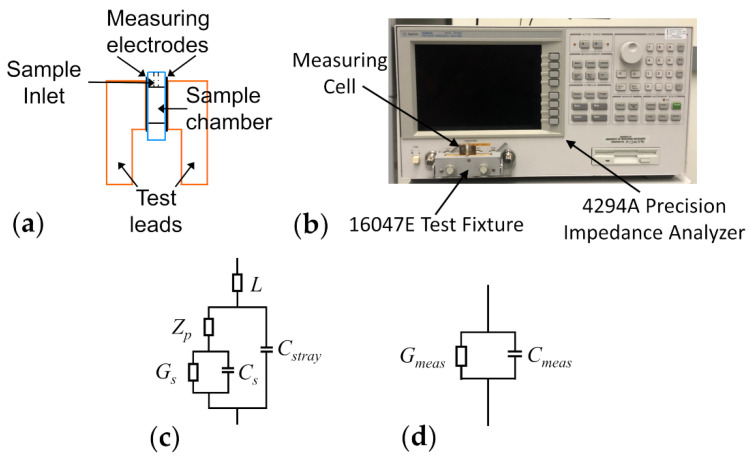
Depiction of the measuring cell and its equivalent circuit. (**a**) Schematic of the parallel-plate capacitor measuring cell. (**b**) Photo of the dielectric spectroscopy measurement setup. (**c**) Electrical circuit describing the measuring cell together with the sample. The sample conductance and capacitance, $G_{s}$ and $C_{s}$, respectively, appear in series with the impedance characterizing the electrode polarization ($Z_{p}$). This combination is in parallel with the stray capacitance ($C_{stray}$) of the measuring electrodes and in series with the inductance (L) of the copper leads. (**d**) Equivalent electrical circuit, consisting of a parallel combination of the measured conductance and capacitance ($G_{meas}$ and $C_{meas}$). These are the values obtained from the measurement apparatus and modelled using Equation (8).

Dielectric measurements of yeast cell samples were performed using an Agilent 4294A precision impedance analyzer (Agilent Technologies, Santa Clara, CA, USA). The samples were placed inside a parallel-plate capacitor measuring cell, the schematic of which has been illustrated in Figure 1a. The electrical leads of the measuring cell were connected to the impedance analyzer via a 16047E test clip fixture (Agilent Technologies, Santa Clara, CA, USA) and the equivalent parallel capacitance and conductance of samples housed in the measuring cell were measured over the frequency range of 40 Hz–110 MHz (see Figure 1b for an image of the measurement apparatus). Prior to every experiment, the impedance analyzer was calibrated, for the purpose of removing inductances arising from the copper leads of the measuring cell from the measured data, using the open/short compensation procedure in accordance with the Agilent 16047E instruction manual. Once the calibration was completed, the measuring cell remained fixed in the 16047E for the duration of the experiment. Any additional uncompensated inductance or capacitance effects introduced via the measuring cell were corrected for in the equivalent circuit used to interpret the measurement data (see Section 2.3). 

Sample solutions were injected, via a syringe needle, into one of two sample inlet/outlet holes bored into a Plexiglas slab making up the body of the measuring cell. Immediately after being entered into the sample chamber, the measurements were carried out upon the sample so as to avoid cell sedimentation within the sample chamber. After electrical measurements were carried out, yeast cell samples were removed from the sample compartment using the suction provided by the syringe needle. The removed sample was immediately centrifugated and the supernatant collected and saved for later electrical measurements using the same measuring cell. Between measurements of different yeast cell suspensions, the sample compartment was rinsed with a liquid similar in conductivity to the suspending medium of the cells (i.e., 20 mM KCl). After the dielectric measurements of all variants of cellular suspensions were taken, the dielectric properties of the corresponding supernatant samples were measured in the exact same manner. All measurements were performed on samples that were allowed to equilibrate to room temperature, which varied from 19.2 to 20.4 °C, depending on the day of experiment. The actual measured temperature was taken into account when choosing the conductivity and permittivity of reference samples, i.e., deionized water and 20 mM KCl, which were measured for the purpose of calibrating the instrument (see Section 2.3 below).

#### 2.2.2. Fluorescence Microspectroscopy

Fluorescence images in the presence of D-Luciferin of yeast cells lacking fluorescently labelled Ste2 were acquired using a spectrally-resolved two-photon optical microspectroscope that was equipped with a line-scan excitation module, as described previously [58]. The microscope consisted of a tunable femtosecond laser (MaiTai^TM^, Spectra Physics, Santa Clara, CA, USA), an inverted microscope (Nikon Eclipse Ti^TM^, Nikon Instruments Inc., Melville, NY), an infinity-corrected, plan apochromat, oil immersion objective (100×, NA = 1.45; Nikon Instruments Inc., Melville, NY, USA), an iXon X3 electron multiplying CCD (Andor Technology, Belfast, UK) and an OptiMiS scanning/detection head (Aurora Spectral Technologies, Grafton, WI, USA). Fluorescence images of yeast cells were acquired using an excitation wavelength of 740 nm and an integration time of 35 ms per pixel. The back-propagating fluorescence emitted by the portion of the sample exposed to the focal volume of the line-shaped excitation beam was collected by the objective and projected onto the cooled electron multiplying CCD (EMCCD). Prior to striking the EMCCD array, the back emitted fluorescence passed through a transmission grating, which separated the emission into its spectral components in the linear dimension orthogonal to the direction of the excitation line. The output of each excitation scan resulted in a set of microspectroscopic images that contained 200 different wavelength channels of ~1 nm bandwidth; the size of the image for each emission wavelength channel was 440 × 300 pixels.

#### 2.2.3. Differential Interference Contrast Measurements

A Leica^TM^ confocal laser scanning microscope was used for imaging the cells in differential interference contrast (DIC) mode. A 100× oil immersion objective with a NA = 1.3 was used. Approximately 10 transmission images were taken for each sample of cells, with each transmission image consisting of ~15 cells. The diameter of each of the cells in an image was measured in two perpendicular directions, and the volume for each cell was calculated assuming a prolate ellipsoidal shape for the cells. The smaller of the two measured diameters for each cell was used as the equatorial diameter in the cell volume calculation. A single radius was then computed for each cell using the measured volume for the corresponding cell, and these values were averaged over entire cell populations to obtain an average cell radius for each type of cell sample. A similar calculation was computed for the size of the vacuolar compartments contained within each cell. 

### 2.3. Simulation of the Measured Dielectric Data

#### 2.3.1. Electrical Model of Yeast Cells

The accumulation of charge at the interface between electrically conducting and insulating compartments of complex biological cells, i.e., interfacial polarization, is the dominant mechanism influencing the frequency-dependent dielectric spectra of biological cells exposed to alternating electric fields in the radiofrequency range. The plasma membrane encloses the entire cell and the membranes enclosing interior organelles within the cell are examples of insulating regions, while the cytoplasm and interiors of the organelles as well as the electrolytic suspending medium represent examples of conducting compartments within the measurement region. Furthermore, yeast cells also possess a rather rigid layer located outside of the cell membrane, known as the cell wall. The dielectric properties of the cell wall are different from those of the outer suspending medium, so a small dielectric dispersion arises in the frequency regime near 10 MHz due to interfacial polarization at the boundary of the wall. Finally, the dielectric properties of yeast have been shown to be adequately simulated using a model that assumes them to be spherical in shape [49,50]. Therefore the dielectric spectra of a yeast cell exposed to an alternating electric field in the radiofrequency range can be simulated using what is known as the spherical three-shell model [50]. As the additional dielectric dispersion due to the cell wall is small and overlaps significantly in frequency with the dispersion due to the vacuolar membrane, and as the third shell adds morphological and electrical parameters to the cell model, the addition of a third shell to the electrical model may become cumbersome in application. However, in this study, particular attention is paid to the electrical properties of the region near this outermost shell—i.e., the near exterior to the cell where the Ste2/α-factor interaction occurs; thus, it is advantageous to incorporate this additional shell into the model used for simulation of the yeast cell dielectric properties, as laid out below. 

The dielectric properties of a suspension of homogeneous spherical particles suspended in a medium with complex permittivity, $\varepsilon_{a}^{*}=\varepsilon_{a}+j\omega \kappa_{a}$, and subjected to an external alternating electrical field of angular frequency, ω, can be expressed as [59,60]:

(1) $$\varepsilon_{s}^{*}=\varepsilon_{a}^{*}\cdot \frac{\left(1+2\cdot v\right)\cdot \varepsilon_{c}^{*}+2\cdot \left(1-v\right)\cdot \varepsilon_{a}^{*}}{\left(1-v\right)\cdot \varepsilon_{c}^{*}+\left(2+v\right)\cdot \varepsilon_{a}^{*}},$$

where $\varepsilon_{s}^{*}=\varepsilon_{s}+j\omega \kappa_{s}$ and $\varepsilon_{c}^{*}=\varepsilon_{c}+j\omega \kappa_{c}$ are the equivalent complex permittivity of the suspension of particles and of the individual particles, respectively, while $v=\frac{volume\;of\;cells}{volume\;of\;suspension}$ is the percentage of volume the cells themselves take up within the entire suspension. The individual particles are represented as a shelled sphere (plasma membrane covered cell) covered by another shell (cell wall). The complex permittivity of the individual particles, $\varepsilon_{c}^{*}$, then presents frequency dependence and is given by:

(2) $$\varepsilon_{c}^{*}=\varepsilon_{w}^{*}\cdot \frac{\left(1+2\cdot v_{w}\right)\cdot \varepsilon_{p}^{*}+2\cdot \left(1-v_{w}\right)\cdot \varepsilon_{w}^{*}}{\left(1-v_{w}\right)\cdot \varepsilon_{p}^{*}+\left(2+v_{w}\right)\cdot \varepsilon_{w}^{*}},$$

where $v_{w}={\left(1-\frac{d_{w}}{R+d_{w}}\right)}^{3}$, R is the radius of the cell, $d_{w}$ is the thickness of the outermost shell (cell wall), $\varepsilon_{p}^{*}$ the complex permittivity of the shelled sphere, and $\varepsilon_{w}^{*}=\varepsilon_{w}+j\omega \kappa_{w}$ is the equivalent complex permittivity of the cell wall.

The complex permittivity of the shelled sphere, $\varepsilon_{p}^{*}$, is given by:

(3) $$\varepsilon_{p}^{*}=\varepsilon_{cm}^{*}\cdot \frac{\left(1+2\cdot v_{cm}\right)\cdot \varepsilon_{i}^{*}+2\cdot \left(1-v_{cm}\right)\cdot \varepsilon_{cm}^{*}}{\left(1-v_{cm}\right)\cdot \varepsilon_{i}^{*}+\left(2+v_{cm}\right)\cdot \varepsilon_{cm}^{*}},$$

where $v_{cm}={\left(1-\frac{d_{cm}}{R}\right)}^{3}$, $d_{cm}$ is the plasma membrane thickness, and $\varepsilon_{cm}^{*}=\varepsilon_{cm}+j\omega \kappa_{cm}$ represents the complex permittivity of the plasma membrane. The parameter $\varepsilon_{i}^{*}$ represents the complex permittivity of the interior region of the cells. As the interior of yeast cells contains membrane bound organelles, $\varepsilon_{i}^{*}$ also depends on frequency. To account for the presence of (more or less concentric) organelles, such as the vacuole in the case of yeast, $\varepsilon_{i}^{*}$ is written as:

(4) $$\varepsilon_{i}^{*}=\varepsilon_{cp}^{*}\cdot \frac{\left(1+2\cdot v_{o}\right)\cdot \varepsilon_{o}^{*}+2\cdot \left(1-v_{o}\right)\cdot \varepsilon_{cp}^{*}}{\left(1-v_{o}\right)\cdot \varepsilon_{o}^{*}+\left(2+v_{o}\right)\cdot \varepsilon_{cp}^{*}},$$

where $\varepsilon_{cp}^{*}=\varepsilon_{cp}+j\omega \kappa_{cp}$ represents the complex permittivity of the cytoplasm, $\varepsilon_{o}^{*}$ the complex permittivity of the intracellular organelle, and $v_{o}$, given by ${\left[\frac{R_{o}}{R-d_{cm}}\right]}^{3}$, stands for the fractional volume the organelle occupies inside the cell, where $R_{o}$ is the radius of the vacuole. As the organelle is covered by a membrane (and is thereby inhomogeneous), $\varepsilon_{o}^{*}$ is frequency-dependent and expressed by:

(5) $$\varepsilon_{o}^{*}=\varepsilon_{om}^{*}\cdot \frac{\left(1+2\cdot v_{om}\right)\cdot \varepsilon_{io}^{*}+2\cdot \left(1-v_{om}\right)\cdot \varepsilon_{om}^{*}}{\left(1-v_{om}\right)\cdot \varepsilon_{io}^{*}+\left(2+v_{om}\right)\cdot \varepsilon_{om}^{*}},$$

where $\varepsilon_{om}^{*}=\varepsilon_{om}+j\omega \kappa_{om}$ and $\varepsilon_{io}^{*}=\varepsilon_{io}+j\omega \kappa_{io}$ are the complex permittivity of the organelle membrane and interior of the organelle, respectively, $v_{om}={\left(1-\frac{d_{om}}{R_{0}}\right)}^{3}$, and $d_{om}$ is the thickness of the vacuolar membrane.

In the formulation presented above, the electric field around any given cell has been assumed to take on the value of the average electric field over the whole space between the electrodes. For highly concentrated suspensions (ν>0.10), the electric field sensed by a cell is also influenced by neighboring cells. Therefore, to properly simulate the dielectric measurements of cell suspensions used in this study (where ν was typically 0.15 or greater), interactions between individual particles must be taken into account. The effective medium theory (EMT) developed by Bruggeman [61] and later Hanai [62,63] extends the usability of the equations presented above to volume fractions up to ν=0.70. The Bruggeman–Hanai theory starts from a dilute suspension of particles, possessing a complex permittivity described by Equation (1), and proceeds to add infinitesimal amounts of particles to this dilute suspension. From this algorithm, a differential equation for the equivalent permittivity, $\varepsilon_{s}^{*}$, was obtained, which was numerically integrated according to a method described previously [64] in order to obtain the complex permittivity of the suspension.

#### 2.3.2. Correction for Electrode Polarization

As stated in Section 2.2.1, the measuring apparatus introduced a number of artefacts to the capacitance and conductance measurements. Therefore, the measured values were expected to include the conductance and capacitance of the sample, alongside contributions from the electrode polarization, the stray capacitance at the edges of the platinum plates, as well as a residual inductance (*L*) from the test leads of the measuring cell. These contributions to the measured electrical properties were all incorporated into the equivalent circuit used to simulate the measured data. In this manner, the intrinsic dielectric properties of the yeast samples under investigation could be accurately extracted from the measured data and further examined.

A well-known artefact inherent to the types of dielectric measurements described in this manuscript is caused by the accumulation of charge at the interface between the measuring electrodes and the electrolytic sample, commonly referred to as electrode polarization (EP) [65]. The EP can cause major distortions in the measured dielectric spectra—e.g., a steep increase in the measured permittivity of the sample occurs in the frequency range below 100 kHz due to EP. The most common method for removing EP is to coat the measuring electrodes with a rough layer of platinum black [65]. While this treatment of the electrodes with platinum black greatly reduces the effect of electrode polarization by ~2 orders of magnitude, the effect of EP cannot be eliminated entirely. Thus, further steps, have to be taken in order to properly separate the contribution of EP to the measured spectra from the contribution of the actual sample [66,67,68]. The contribution of electrode polarization to the equivalent circuit describing the measured data (Figure 1c) has been found to be accurately modelled by a constant phase angle (CPA) element, in series with the sample dielectric properties [67,69,70]. The CPA element is described by the following equation:

(6) $$Z_{p}=a^{-1}{\left(j\omega \varepsilon_{o}\right)}^{-b},$$

where a is a parameter related to a number of factors, most notably the electrical properties of the sample, while the parameter b is solely dependent on the surface characteristics (e.g., roughness) of the electrode [70] and can take on values in the range of [0,1]. By combining Equation (1) with Equation (6) and assuming a serial relationship between EP and sample contributions, the total admittance, Y, of the sample chamber region can be written as:

(7) $$Y={\left(Z_{p}+\frac{1}{jk_{cell}\omega \varepsilon_{o}\varepsilon_{s}^{*}}\right)}^{-1},$$

where $\varepsilon_{s}^{*}$ is computed from Equation (1), $\varepsilon_{o}$ is the permittivity of free space equal to 8.854 × 10^−12^ F/m, and $k_{cell}$ is a geometrical constant associated with the physical dimensions of the parallel-plate capacitor. 

There exists a stray capacitance, $C_{stray}$*,* which also contributes to the measured data. The stray capacitance arises as the electric field distribution reaches further than the sample chamber dimensions and penetrates the surrounding Plexiglass base. Therefore, to properly incorporate the stray capacitance in the equivalent circuit, $C_{stray}$ was placed in parallel with the admittance, Y, of the sample/EP series combination (Equation (7)). Furthermore, even though it was partly corrected for, a small residual inductance, *L*, associated with the test leads of the measuring cell still influenced the measurements. This circuit component was placed in series with the series combination of the sample and electrode polarization equivalent circuit components (Figure 1c). Therefore, the total measured admittance (Figure 1d), $Y_{measured}=G_{meas}+j\omega C_{meas}$, was modelled as:

(8) $$Y_{theo}=\frac{Y+j\omega C_{stray}}{1+j\omega L\left(Y+j\omega C_{stray}\right)},$$

The measured conductivity, $\kappa_{meas}$, was obtained by dividing $G_{meas}$ by $k_{cell}$; the measured relative permittivity was obtained by dividing $C_{meas}$ by $k_{cell}$ and and normalizing to the free space permittivity. The values for $k_{cell}$ as well as the $C_{stray}$ and *L* circuit components were determined from measurements of liquid samples with well-known dielectric properties—i.e., deionized water and 20 mM KCl solutions [71]. Simulation of dielectric measurements obtained from yeast cells using Equation (8) was performed by keeping the *L* and $C_{stray}$ values fixed, and adjusting a number of the remaining parameters (see Section 2.3.3 below) used in Equations (1)–(6), such that the discrepancy between the measured data and theoretical simulation was minimized. The goodness-of-fit was quantified by summing the discrepancy between the measured and theoretical permittivity and conductivity values at each measured frequency ω, as given by the following standard fitting residual (or cost) function:

(9) $$Res={\left\{\underset{}{\sum }{\left[1-\frac{\log\left(\varepsilon_{theo}\right)}{\log\left(\varepsilon_{meas}\right)}\right]}^{2}+\sum {\left[1-\frac{\kappa_{theo}}{\kappa_{meas}}\right]}^{2}\right\}}^{0.5}\cdot 100.$$

#### 2.3.3. Dielectric Data Fitting Algorithms and Procedures

Minimizing the residual function displayed in Equation (9) can be challenging in practice due to the large number of parameters needed to implement the three-shell model. A brute-force search for the residual minimum is computationally unrealistic due to the extremely large parameter space that must be tested. Therefore, one must rely on an approximation algorithm, such as an iterated improvement algorithm, to estimate the best solution in a realistic time frame. Iterated improvement algorithms start with some initial configuration of parameters and sample a neighboring configuration in the parameter space. The neighboring configuration is accepted as the new solution only if it has a lower value of Res than the previous configuration. This process is repeated until a point is reached in parameter space in which all neighboring configurations have higher Res values. However, in this approach, there is no way of avoiding local minima—i.e., locations in parameter space in which all neighboring configurations have higher Res values but do not represent the set of parameters which gives the absolute global minimum of Res. 

For this reason, we have utilized the Simulated Annealing (SA) algorithm [51,72] to locate a good approximation to the global minimum of the residual function, Res, given the large multidimensional space associated with the three-shell model. Briefly, the SA algorithm begins with an initial configuration of parameters, *i*, and slightly perturbs this configuration to a random neighboring configuration, *j*. If $Res_{j}$ < $Res_{i}$, then the new configuration is accepted. If $Res_{j\;}$> $Res_{i}$, then the new configuration is not necessarily rejected, but is accepted with a probability of $e^{\frac{-\left(Res_{j}-Res_{i}\right)}{kT}}$, where k is the Boltzmann constant, and T is an iterative parameter used during the implementation of the SA algorithm. Initially, the value for the T parameter is set to a high value (*T* = 15) such that movements from configurations with low Res values to high Res values are accepted at a high rate and hence the movement out of a local minimum is readily allowed. As the algorithm proceeds, the value of T is systematically decreased, such that during the final stages of the algorithm, only “downhill” movements within the configuration space, i.e., changes to the parameters that result in a decrease in Res, are accepted. The SA algorithm ingeniously combines aspects of iterated improvement algorithms with randomization techniques to avoid the problem of falling into local minima by accepting, with a nonzero probability, movements within the parameter configuration space which lead to an increase in the value of Res. Furthermore, the final solution is independent of the initial parameter configuration, adding objectivity to the data analysis process. 

**Figure 2:**
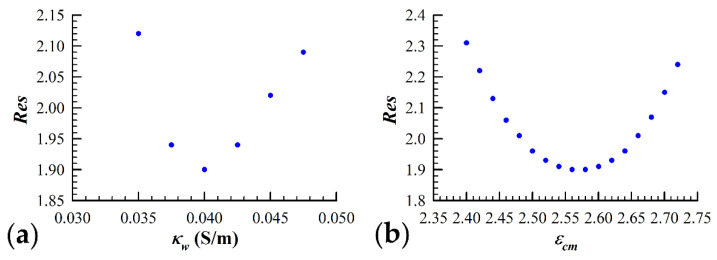
Validation of the three-shell model applicability. The fitting procedure, as described in Section 2.3.3, was performed by iterating a number of key model parameters to find the global minimum fit of the spherical three-shell model to the measured data. (**a**) The conductivity of the cell wall, $\kappa_{w}$, was iterated in increments of 0.0025 S/m until the Res values obtained for a given data set were greater than 10% of the minimum value obtained. (**b**) Using the value for $\kappa_{w}$ which resulted in the simulation of the measured data with the lowest fitting residual ($\kappa_{w}$ = 0.04 S/m), the iteration process was repeated by changing the permittivity of the cell membrane ($\varepsilon_{cm}$) in increments of 0.02. As it can be seen in the figure, there exists a unique set of parameters that give the “best fit” to the measured data.

Another concern associated with simulating yeast cell dielectric data with the three-shell model is that the solution corresponding to the lowest attainable Res value is degenerate. This potential issue arises due to the fact that the contribution to the measured spectrum from the cell wall (third shell) overlaps significantly with the interior vesicle contribution. In order to check for the uniqueness of the parameter configuration space in terms of the fitting residual minimum, we were required to implement a second iterative process in conjunction with the SA algorithm. We focused on the uniqueness of a couple of key three-shell model parameters, $\varepsilon_{cm}$ and $\kappa_{w}$, which are strongly linked through their relationship to the value of the volume fraction, v. The height of the permittivity plateau is largely determined by both v and $\varepsilon_{cm}$. The low frequency (<100 kHz) conductivity data are influenced heavily by the volume fraction and the conductivity of the cell wall, $\kappa_{w}$. Special attention was given to these two relationships due to the importance of the plasma membrane dielectric properties in this study. The SA algorithm was executed with the value of $\kappa_{w}$ held at a fixed value, and the minimum Res value for that particular $\kappa_{w}$ was found. Once a minimum was reached, the parameter values were recorded along with the Res value. The value of $\kappa_{w}$ was then altered by ~8%, and the SA algorithm was similarly executed until a minimum Res value was once again reached. Figure 2a visually represents this process, as the value of fitting residual reached using the SA algorithm is plotted against the corresponding value of $\kappa_{w}$. As is evident from the figure, there clearly exists a single value of $\kappa_{w}$ which best simulates the measured data. Once the value of $\kappa_{w}$ was determined, the process was repeated except with $\varepsilon_{cm}$ as the iterated parameter and $\kappa_{w}$ held fixed to the value determined from the first iterative procedure. Figure 2b shows that a minimization of the fitting residual can only be accomplished with a single value of $\varepsilon_{cm}$, which we assume to be the permittivity of the plasma membrane of the yeast cells under study.

Finally, a number of the fitting parameters were ascertained using secondary measurements of the given cell suspension and held fixed throughout the data fitting procedure. Parameters related to the size of the cells, R and $R_{0}$, were determined via wide field microscopy measurements performed upon the cells immediately after the dielectric measurements were finished. The values for R and $R_{0}$ were measured for all samples on each day of the experiment. The observed standard deviation of the average radius measurements from experiment to experiment was much smaller than the distribution of sizes within a given population of cells. Therefore, a single value for both cellular and vacuolar radius was calculated from the size measurements obtained over all experiments (n = 6). These two averages, *R* = 1.65 µm and $R_{0}$ = 1.25 µm, were then used for each cellular population throughout the data fitting procedure. The size of the plasma and vacuolar membranes were also held fixed at $d_{cm}$ = $d_{om}$ = 2.5 nm, while the cell wall thickness was set to be $d_{w}$ = 0.25 μm, values that were obtained from previously published reports [73]. The extracellular medium permittivity and conductivity, $\varepsilon_{a}$ and $\kappa_{a}$ respectively, were also determined prior to the fitting procedure by immediately centrifuging the cells after the dielectric measurements were performed, collecting the supernatant, and measuring the dielectric properties of the supernatant. The measured spectrum of the supernatant was then fit with Equation (8) and *ν* = 0, leaving only *a*, *b*, $\varepsilon_{a}$, and $\kappa_{a}$ as fitting parameters. The values obtained for $\varepsilon_{a}$ and $\kappa_{a}$ were then used and held fixed when fitting the spectrum of the corresponding yeast cell suspension. Finally, the conductivity of both the plasma and vacuolar membrane regions, $\kappa_{cm}$ and $\kappa_{om}$, were set to zero for the data fitting process.

## 3. Results and Discussions

### 3.1. Dielectric Measurements of Ste2-Expressing Yeast Cells Exposed to α-Factor

**Figure 3:**
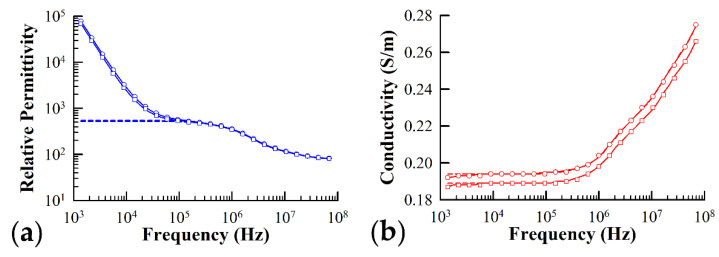
Typical plots of the relative (**a**) permittivity and (**b**) conductivity of yeast cells expressing the sterile2 α-factor receptor protein in the presence (circles) and absence (squares) of the α-factor ligand. The theoretical best fit to the measured data predicted by the three-shell model presented in the text is represented by the solid lines. The dashed lines represent the theoretical fit with the correction due to the electrode polarization subtracted off. Fitting parameters used in simulation of measured spectra for cells in the presence of α-factor: $d_{cm}$ = 2.50 nm, $d_{om}$ = 2.50 nm, R = 1.65 µm, $R_{0}$ = 1.25 µm, v = 0.17, $\varepsilon_{a}$ = 78.0, $\kappa_{a}$ = 0.25 S/m, $\varepsilon_{w}$ = 90.7, $\kappa_{w}$ = 0.04 S/m, $\varepsilon_{cm}$ = 2.53, $\kappa_{cm}$ = 0 S/m, $\varepsilon_{cp}$ = 46.5, $\kappa_{cp}$ = 0.27 S/m, $\varepsilon_{om}$ = 12.4, $\kappa_{om}$ = 0 S/m, $\varepsilon_{io}$ = 25.0, $\kappa_{io}$ = 1.43 S/m, a = 1.13 × 10^5^, b = 0.79. Fitting parameters used in simulation of measured spectra for cells not exposed to α-factor: $d_{cm}$ = 2.50 nm, $d_{om}$ = 2.50 nm, R = 1.65 µm, $R_{0}$ = 1.25 µm, v = 0.16, $\varepsilon_{a}$ = 78.0, $\kappa_{a}$= 0.24 S/m, $\varepsilon_{w}$ = 90.7, $\kappa_{w}$ = 0.04 S/m, $\varepsilon_{cm}$ = 2.46, $\kappa_{cm}$ = 0 S/m, $\varepsilon_{cp}$ = 62.9, $\kappa_{cp}$ = 0.33 S/m, $\varepsilon_{om}$ = 11.9, $\kappa_{om}$ = 0 S/m, $\varepsilon_{io}$=25.0, $\kappa_{io}$ = 1.00 S/m, a = 1.83 × 10^5^, b = 0.82.

Figure 3 illustrates the typical frequency dependence of both the permittivity (Figure 3a) and conductivity (Figure 3b) of yeast cells expressing Ste2 in the presence and absence of α-factor. The solid lines in the graph represent the best fit simulation of the data using Equation (8) and the data fitting algorithms detailed in Section 2.3.3, while the dashed lines represent the theoretical best fit with the electrode polarization contribution subtracted from the sample admittance, Y. 

The set of fitting parameters extracted for each sample were those which resulted in the smallest difference, as judged by the fitting residual given in Equation (9), between the measured dielectric spectra and corresponding simulated curves. The difference, $\delta_{m}$, between the fitting parameters extracted from dielectric spectra obtained from cells which had been exposed to alpha factor (+α) and those which had not (−α) was computed for each pair of samples. “Pairs” of samples were those which were prepared as one cell suspension right up until the final step before dielectric measurements, at which point the single cell suspension was split into the pair of samples, with α-factor added to one of the volumes and not added to the other (see Section 2.1 for a more detailed description of the sample preparation). The individual $\delta_{m}$ values calculated for each pair of samples was then averaged over all measurements, according to the following equation:

(10) $$\langle \delta_{m}\rangle =\overset{n}{\underset{i=1}{\sum }}\frac{m_{i,+\alpha }-m_{i,-\alpha }}{n},$$

where m represents a particular fitting parameter, e.g., $\varepsilon_{cm}$, and *i* is the index for a particular sample pair. The value of $\langle \delta_{m}\rangle$ was calculated for a number of different fitting parameters used in the three-shell model, which are associated with the electrical properties of particular regions of the cells under study, i.e., $\varepsilon_{w}$, $\kappa_{w}$, $\varepsilon_{cm}$, $\varepsilon_{cp}$, and $\kappa_{cp}$, and listed in Table 1. Also listed in Table 1 is the result of a paired Student’s *t*-test performed between each of the analogous parameters of paired samples. The *t*-test *p* value presented in Table 1 is the probability that the difference in the means of the two values occurs by chance. As is evident from the table, the dielectric properties of a number of cellular compartments, i.e., permittivity of the plasma membrane and both the permittivity and conductivity of the cytoplasm, change by a statistically significant amount (i.e., *p* < 0.05) after treating the cells with α-factor.

The statistically significant difference in the plasma membrane permittivity between the two cell samples was, to a certain extent, expected because the α-factor ligand is known to bind to the Ste2 in the outer cellular region [74], thereby directly disturbing the region of the cells near the plasma membrane. A little more surprising was the significant change occurring to the cytoplasm electrical phase parameters ($\varepsilon_{cp}$ and $\kappa_{cp}$) after adding α-factor to the cell suspension. The difference in $\varepsilon_{cp}$ and $\kappa_{cp}$ between the two samples indicates that the presence of the α-factor in the cell suspension also causes an alteration in the cytoplasm electrical makeup. It is well-known that α-factor internalization occurs through binding to Ste2 followed by internalization of the complex. The process is followed by decoupling of alpha factor and quality check of the receptors in view of their possible relocation to the plasma membrane or destruction of the receptor if it fails the quality check. The fact that dielectric spectroscopy was able to detect changes in the cytoplasm electrical properties caused by such biochemical processes is remarkable. 

### 3.2. Dielectric Measurements of Yeast Cells Lacking Ste2 Receptors

The dielectric data acquisition and analysis protocol (see Section 2.2.1 and Section 2.3) was performed upon Ste2Δ yeast cells that lacked expression of Ste2 but were otherwise prepared in the same manner as the Ste2-expressing cells. As it was computed for the Ste2-expressing cells described in Section 3.1, the value of $\langle \delta_{m}\rangle$ was calculated for paired sets of measurements obtained from Ste2Δ cell suspensions for a number of different fitting parameters. The various values of $\langle \delta_{m}\rangle$ are given in Table 2 along with the results of a paired Student’s t-test performed for each of these parameters. As seen, the value of $\varepsilon_{cm}$ determined for Ste2Δ cells remains unchanged upon addition of α-factor to the suspending medium of the cells (i.e., $\langle \delta_{\varepsilon_{cm}}\rangle =0$). This result confirms that the change in $\varepsilon_{cm}$ observed when the Ste2-expressing cells were exposed to α-factor is caused by the actual binding of the α-factor to the Ste2 receptors docked at the plasma membrane – an encouraging result which displays the sensitivity of dielectric spectroscopy to very specific activities of sub-cellular regions. By this standard, the current experiment, which was meant to serve as a control, fulfilled its role quite well. 

Quite surprisingly, however, the dielectric properties of the cytoplasmic region of the Ste2Δ cells, i.e., $\varepsilon_{cp}$ and $\kappa_{cp}$, also showed significant changes following the addition of the α-factor to the cell suspension. This suggested that the presence of the α-factor in the external medium causes a noticeable reaction by the cells which is affecting their internal dielectric properties. As seen in Table 2, the changes detected in the Ste2Δ cytoplasmic properties are rather large in comparison to the variability of the data. Specifically, the value of $\langle \delta_{\varepsilon_{cp}}\rangle$ is 1.5 times the standard deviation of the $\delta_{\varepsilon_{cp}\;}$ values; an even greater contrast is seen for the conductivity of the cytoplasm, where $\langle \delta_{\kappa_{cp}}\rangle$ is 2.5 times the standard deviation of the $\delta_{\kappa_{cp}\;}$ values. In fact, it appears that the changes occurring to $\varepsilon_{cp}$ and $\kappa_{cp}$ in the Ste2Δ cells is even larger than the change seen in those parameters for the Ste2-expressing cells, as evidenced by the larger ratios between average and the standard deviation of the $\delta_{m\;}$ values as well as the smaller *p*-value. One potential explanation for this large change in the cytoplasmic dielectric properties of the cells exposed to α-factor is that α-factor is able to cross the cell membrane without relying on receptor-mediated endocytosis. This alternative mechanism for internalization would explain the larger changes seen in $\varepsilon_{cp}$ and $\kappa_{cp}$ when Ste2 is absent from the cell membrane, as α-factor is no longer impeded by binding to Ste2 at the membrane, and is therefore free to enter the cell via this hypothetical alternative internalization mechanism. In contrast, the presence of tailless Ste2 on the membrane in the Ste2-expressing cells would impede a portion of the α-factor molecules from entering the cell due to their binding to the plasma membrane located Ste2. 

The idea that the ligand could cross the cell membrane without needing the mechanism of receptor-mediated endocytosis is not without precedent. While once thought to only occur at the plasma membrane, biologically relevant interactions between GPCRs and their cognate ligands have been observed in internal membranes [75], which resulted in the GPCR interacting with a G protein to initiate a signaling cascade [76]. The internalization of the ligand and activation of GPCRs located in subcellular locations can occur in a variety of ways other than through receptor-mediated endocytosis; for example, ligands can be transported through a channel or pores [77] or even pass into the cell via diffusion [78]. Certain GPCR ligands are known to be membrane-soluble and can bind to their respective GPCR from within the lipid bilayer or even in the interior of the cell [79]. The ability of the ligand to penetrate the membrane depends on various factors, including the polarity of the ligand itself, the composition of the membrane [79], e.g., the percentage of cholesterol content, as well as the intracellular and/or extracellular pH. 

Below, we explore this hypothesis in further detail to determine whether or not the cellular cytoplasmic dielectric property changes are directly related to a specific cellular process involving the internalization of the α-factor or related to simple environmental medium changes causing internal reactions from the cell. 

### 3.3. Testing the Effect of Osmotic Pressure Variation on the Cytoplasm Dielectric Properties

One possible explanation for the changes in the cytoplasm dielectric properties of both Ste2Δ and Ste2-expressing cells upon exposure to α-factor is the change in osmotic pressure difference across the cell membrane due to the addition of the ligands to the cellular suspending medium. If the change in osmotic pressure is large enough, the osmosis of water from the cell cytosol to the suspending medium (which is exterior to the cell) could affect the dielectric measurements in a number of ways: the cytoplasm conductivity would increase due to a higher ionic concentration, and the size of the cells would decrease because of the loss of water. The extent to which these effects influenced the dielectric measurements of cells exposed to α-factor was studied in more detail by two different sets of measurements designed to probe each of the hypothesized effects separately. 

**Figure 4:**
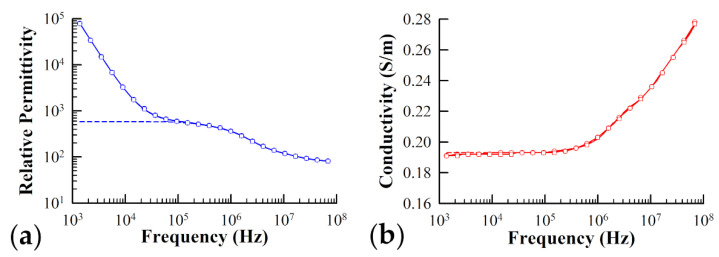
Typical plots of the (**a**) relative permittivity and (**b**) conductivity of yeast cells suspended in a 0.02 M KCl (squares) and yeast cells suspended in a 0.02 M KCl solution containing sorbitol (circles). The concentration of sorbitol in solution was 250 μM in order to replicate the osmotic pressure felt by yeast cells exposed to the same concentration of α-factor. The theoretical best fit to the measured data predicted by the three-shell model presented in the text is represented by the solid lines. The dashed lines represent the theoretical fit with the correction due to the electrode polarization subtracted off. Fitting parameters used in simulation of measured spectra for cells in the presence of sorbitol: $d_{cm}$ = 2.50 nm, $d_{om}$ = 2.50 nm, R = 1.65 µm, $R_{0}$ = 1.25 µm, v = 0.18, $\varepsilon_{a}$ = 78.0, $\kappa_{a}$ = 0.26 S/m, $\varepsilon_{w}$ = 83.5,$\;\kappa_{w}$ = 0.035 S/m, $\varepsilon_{cm}$ = 2.52, $\kappa_{cm}$ = 0 S/m, $\varepsilon_{cp}$ = 39.8, $\kappa_{cp}$ = 0.25 S/m, $\varepsilon_{om}$ = 11.8, $\kappa_{om}$ = 0 S/m, $\varepsilon_{io}$ = 60.1, $\kappa_{io}$ = 1.65 S/m, a = 1.41 × 10^5^, b = 0.81. Fitting parameters used in simulation of measured spectra for cells not exposed to sorbitol: $d_{cm}$ = 2.50 nm, $d_{om}$ = 2.50 nm, R = 1.65 µm, $R_{0}$ = 1.25 µm, v = 0.18, $\varepsilon_{a}$ = 78.0, $\kappa_{a}$ = 0.26 S/m, $\varepsilon_{w}$ = 86.6, $\kappa_{w}$ = 0.035 S/m, $\varepsilon_{cm}$ = 2.51, $\kappa_{cm}$ = 0 S/m, $\varepsilon_{cp}$ = 38.9, $\kappa_{cp}$ = 0.24 S/m, $\varepsilon_{om}$ = 11.6, $\kappa_{om}$ = 0 S/m, $\varepsilon_{io}$ = 41.2, $\kappa_{io}$ = 1.63 S/m, a = 1.46 × 10^5^, b = 0.81.

In order to test the effect of changing osmotic pressure on the cytoplasm dielectric properties, the dielectric data acquisition and analysis protocol (see Section 2.2.1 and Section 2.3) was repeated upon Ste2Δ yeast cells exposed to the sugar alcohol Sorbitol. The addition of Sorbitol to the cell suspending medium provided a means to increase osmotic pressure the cells experienced in the presence of α-factor, without causing any appreciable physiological response by the cells which would further complicate the dielectric comparison to cells suspended in simple electrolyte. Figure 4 illustrates the typical frequency-dependent behavior of both the permittivity (Figure 4a) and conductivity (Figure 4b) of Ste2Δ yeast cells suspended in an electrolyte solution containing a 250 μM concentration of Sorbitol (circles), as well as Ste2Δ cells suspended in the equivalent electrolyte solution minus the Sorbitol (squares). The solid lines in the figure represent the best fit to the measured data using Equation (8) and the data fitting procedure detailed in Section 2.3.3. The value of $\langle \delta_{m}\rangle$ was calculated for a number of the electrical phase parameters associated with the pertinent compartments of the cells and listed in Table 3. Of course, for these particular measurements, $\langle \delta_{m}\rangle$ reflects a difference in electrical phase parameters measured between pairs of samples, one of which was treated with Sorbitol (rather than α-factor which was the case for the data presented in Section 3.1 and Section 3.2). Similar to Table 1 and Table 2, the result of a Student’s t-test applied to each pair of parameters is also listed in the table. As can be seen from Table 3, no significant change occurred to any of the parameters describing the dielectric properties of both the plasma membrane and cytoplasm regions of the cells upon addition of the Sorbitol to the cellular suspending medium. In particular, the *p* values comparing $\varepsilon_{cm}$, $\kappa_{cp}$, and $\varepsilon_{cp}$ between the two types of samples were extremely high (*p* > 0.9).

The osmotic pressure of a solution is proportional to the sum of the molar concentrations of all constituents of the solution. In the extracellular solution, the dissociated ions of the KCl salt are in the highest concentration (0.02 M), and hence contribute most to the osmotic pressure. In contrast, the concentration of the additional molecules (i.e., Sorbitol or α-factor) within the exterior medium solution is low (~25 × 10^−4^ M). The contribution of the additional molecules to the osmotic pressure on the exterior side of the cell membrane would be two orders of magnitude less than that due simply to the ions of the 0.02 M KCl solution. Therefore, any reaction to this minute difference in pressure exerted upon the membrane due to the sorbitol molecules would result in a small change in ion concentration, and hence internal conductivity, of the cell cytosol. However, the change in internal conductivity sensed by the dielectric measurements performed on both Ste2Δ- and Ste2-expressing samples was 6% and 10%, respectively. The experimental results presented in this section support rejection of the hypothesis that an increase in osmotic pressure was the principal cause for the change in the internal dielectric properties of the yeast cells after adding α-factor to the suspending medium. 

We next tested whether the addition of α-factor to the cell suspending solution caused an appreciable change in size of the cells. Both Ste2-expressing cells and cells devoid of Ste2 (Ste2Δ) were prepared and treated in the same manner as the cells reported on in Section 2.1. DIC measurements were obtained from all four sets of samples in order to test whether the addition of α-factor caused a swelling of the cells due to an external osmotic pressure change. Table 4 lists the value of $\langle \delta_{m}\rangle$ calculated for both the cell and vacuole radius measured for cells suspended in a simple electrolyte solution as well as an electrolyte solution with α-factor added to it. As seen in row 3 of the table, neither the size of the whole cell nor the size of the vacuole compartment of the yeast experiences a significant change upon addition of α-factor to the cellular suspension.

### 3.4. Determining the Extent of Nonspecific Internalization Using the Fluorescent Molecule D-Luciferin

We also wanted to test whether the ligand was internalized by the cell via a specific or nonspecific mechanism. It should be noted that, while it is known that wild-type Ste2 undergoes endocytosis after binding to α-factor, Rohrer et al. and a number of other investigators have demonstrated that cutting the cytoplasmic tails of the Ste2 at or before amino acid number 326 results in the elimination of α-factor endocytosis via binding to Ste2 [80,81,82]. In the present study, the DNA encoding the Ste2 cytoplasmic tail has been removed at amino acid 304 and replaced with the instructions for synthesizing an attached fluorescent protein (either GFP2 or YFP). Hence, the endocytosis inducing mechanism which naturally occurs to the Ste2 upon ligand binding has actually been removed from the Ste2 proteins.

There exists a second form of endocytosis, i.e., pinocytosis, which can be used by the cells to internalize the α-factor (or other molecules) and does not rely on the presence and actions of specific membrane receptors. To test the degree of pinocytosis occurring when particles are suspended in the yeast cell suspension external medium, yeast cells were suspended in a 250 μM solution of D-luciferin for 25 min, washed three times with 100 mM KCl, resuspended in 100 mM KCl, and then imaged using a two-photon optical microspectroscopy system. D-luciferin was chosen because it is both fluorescent and directly excitable in our spectrally resolved two-photon microscope, which allowed us to quantify the cellular intake of D-luciferin, which may occur in the absence of molecule-specific internalization mechanisms 

Using the spectrally resolved two-photon microscope described in Section 2.2.2, we obtained both the intensity of fluorescence emanating from various yeast cells, and the spectral information of this emitted light. The latter allowed us to determine whether the fluorescence emission was due strictly to the fluorescent D-luciferin molecules, as opposed to other molecular species within the cells which possess intrinsic fluorescence, e.g., nicotinamide adenine dinucleotide (NADH) or flavin adenine dinucleotide (FAD). The composite spectrum comprised of signal from these other molecular species which possess intrinsic fluorescence is referred to as “autofluorescence” herein. We found the signal level of D-luciferin detected in cells which had been exposed to D-luciferin was comparable to the inherent autofluorescence being given off by the cells at the excitation wavelength used to excite D-Luciferin (740 nm). Therefore, the emission spectrum from each pixel in the microspectroscopic images was deconvoluted into D-luciferin and autofluorescence components using a least-squares fitting algorithm along with separately determined elementary spectra of each of the fluorescent components, as described elsewhere [54,57,83]. The autofluorescence spectrum was obtained from microspectroscopic measurements of yeast cells not exposed to D-Luciferin, while the D-Luciferin spectrum was obtained from measurements of a 50 μM solution of D-Luciferin. Applying the unmixing procedure to each image pixel resulted in 2D maps of D-luciferin and autofluorescence signal.

**Figure 5:**
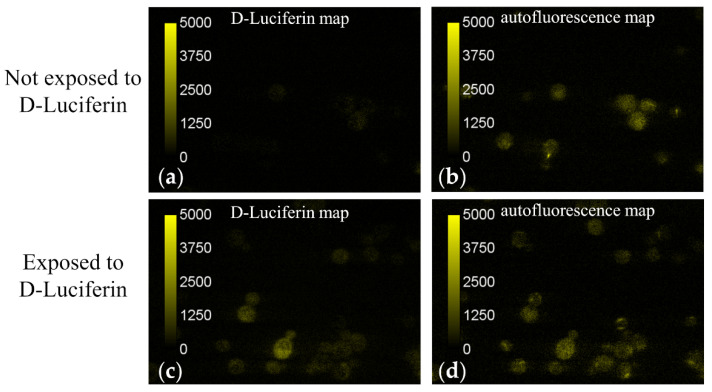
Fluorescence intensity maps of D-Luciferin and autofluorescence signal obtained by unmixing microspectroscopic images of yeast cells before (**a**,**b**) and after (**c**,**d**) exposure to the naturally fluorescent molecule D-luciferin. Intensities are assigned false colors according to their value and a correlation scale is given as an inset. (**a**,**b**) A representative field of view obtained from yeast cells prior to exposure to D-Luciferin is shown for both the unmixed (**a**) D-Luciferin and (**b**) autofluorescence intensity maps. A D-Luciferin component was used in the unmixing, even for the cells not exposed to D-Luciferin, in order to establish a noise baseline for detecting D-Luciferin signal. The mean pixel intensities of the D-Luciferin and autofluorescence signals (n = 148 cells) were 36 ± 153 and 1000 ± 351 counts, respectively. (**c**,**d**) The yeast cells were suspended in a 250 μM solution of D-luciferin for 25 min. The cell/D-luciferin suspension was then centrifuged and washed with 1 mL 100 mM KCl three times before resuspending in 100 μL of 100 mM KCl. The mean pixel intensities of the D-Luciferin and autofluorescence signals (n = 154 cells) were 455 ± 250 and 723 ± 319 counts, respectively. Because the average D-Luciferin signal was larger than the baseline value established for cells not exposed to D-Luciferin, it was determined that there was a small amount of pinocytosis of the D-Luciferin molecules occurring.

Figure 5 depicts typical fields of view of the D-Luciferin and autofluorescence signals obtained by unmixing microspectroscopic images of cells which had been exposed as well as those which had not been exposed to D-Luciferin. Intensity values in these images are assigned false colors according to the scale inset seen in the leftmost region of the image. We found that the autofluorescence spectrum varied slightly from cell to cell. To account for these slight shifts in the autofluorescence spectrum, the unmixing returned a nonzero value for D-luciferin signal for some cells, even in the cells which were not exposed to D-Luciferin prior to imaging (e.g., see Figure 5a). Therefore, measurement of cells not exposed to D-Luciferin also provided us with a baseline noise level of the D-luciferin signal expected from these measurements.

Using the autofluorescence spatial intensity map along with the open source software program Image J, regions of interest (ROIs) were drawn around the outer boundary of each imaged cell. The mean intensity value of the autofluorescence and D-Luciferin signal was then measured for the pixels contained within each ROI. An average intensity value was then computed across all imaged cells for both fluorescence components (i.e., D-Luciferin and autofluorescence) by finding the mean pixel intensity value from each cell. For the cells which had been suspended in a 250 μM solution of D-Luciferin, the average value of the D-Luciferin signal was found to be 455 ± 250 counts (n = 154 cells), which was only slightly less than the average unmixed autofluorescence signal, which was found to be 723 ± 319 counts. In contrast, the baseline noise level of D-Luciferin signal detected by using a D-Luciferin component in the unmixing of cells which had not been subjected to D-Luciferin prior to imaging was 36 ± 153 counts for n = 148 cells, significantly less than the autofluorescence signal found from the same cells, 1000 ± 351. Because the average D-Luciferin signal was significantly more than the baseline level established for cells not exposed to D-Luciferin, it was determined that there was a detectable amount of pinocytosis of the D-Luciferin molecules occurring at a concentration of 250 μM.

The fluorescence data acquisition and analysis of yeast cells exposed to D-luciferin solutions presents proof, by both simple visual inspection and quantification using spectral deconvolution, that the yeast cells are able to internalize solute molecules suspended in the extracellular medium, albeit at a fairly modest rate. The level of D-Luciferin signal detected in cells exposed to D-Luciferin (455 ± 250 counts), was approximately three orders of magnitude less than the mean signal level of a 250 μM solution of D-Luciferin (970,000 counts) obtained using the same imaging conditions. The modest levels of nonspecific endocytosis observed in the images taken of yeast cells exposed to D-Luciferin solutions presents indirect evidence that pinocytosis of α-factor could, at least in part, contribute to the significant changes to the dielectric properties of the cell cytoplasm which were detected in the dielectric measurements. However, this modest level of pinocytosis detected would most likely not fully account for the significant changes seen in the yeast cell cytoplasmic dielectric properties. However, any proposed additional mechanisms for α-factor to penetrate the cell membrane needs further investigation.

## 4. Conclusions

In this work, we have used dielectric spectroscopy to detect the onset of subcellular processes occurring in specific compartments of biological cells. Specifically, we have shown evidence of the binding between a GPCR, the sterile2 α-factor receptor, and its agonist, the α-factor. The permittivity of the yeast plasma membrane increased by a significant amount after adding α-factor to a suspension of the yeast cells expressing Ste2 in their plasma membranes. In contrast, the membrane permittivity of yeast cells not expressing Ste2 remained constant upon exposure to an equivalent concentration of α-factor. 

Furthermore, we have shown that for both the cells expressing and those lacking Ste2, the presence of α-factor in the immediate cell exterior caused a large change in the dielectric properties of the cytoplasm. Modifications to the cytoplasmic tail of the Ste2 expressed by the cells used in these studies is known to inhibit receptor-mediated endocytosis [80], which therefore rules out this mechanism as the reason for the cytoplasmic dielectric property changes. Furthermore, evidence obtained through complementary measurements ruled out the simple effect of osmosis as the reason for these changes. The nonspecific endocytosis mechanism of pinocytosis was also tested as a possible reason for the change in dielectric properties of the cytoplasm. To quantify the extent of pinocytosis the cells were capable of, we imaged cells which had been exposed to the naturally fluorescent molecule D-luciferin and computed the amount of D-luciferin transported into the cell interior. Fluorescence images taken of cells showed a modest amount of D-luciferin internalized by the yeast cells, and therefore we attributed the nonspecific mechanism of pinocytosis, at least in part, to the change in dielectric properties of the cell cytoplasm. However, the rather significant change to the cell cytoplasmic dielectric properties sensed upon addition of α-factor, compared with the small amount of pinocytosis detected, does suggest that another mechanism might also allow for internalization of the α-factor, which would also contribute to the change in cell cytoplasm dielectric properties. 

One potential reason for α-factor to enter the cell and interact with Ste2 internally derives from the fact that many GPCRs which are misfolded and retained in the endoplasmic reticulum can be functionally rescued by binding to certain small molecules known as chaperones [84,85]. Chaperones facilitate proper trafficking of the GPCR to the plasma membrane after promoting the correct folding of the GPCR by introducing stabilizing conformational constraints in the folding pathway. While a large number of known molecular chaperones are nonspecific, it has also been shown that, for certain GPCRs, endogenous agonists can act to both restore membrane trafficking and promote activation of the specific GPCR when it returns to the membrane [86,87]. These receptor-specific chaperones have the benefit of preferentially rescuing a misfolded GPCR in a biased manner. Whether or not α-factor is capable of permeating the cell membrane and acting as a chaperone for Ste2 requires follow up studies using a fluorescently labelled α-factor. We also cannot rule out α-factor interactions with other proteins or ligands within the cell as the reason for α-factor entering the cell. It is known that the presence of histidine in the second position within the α-factor sequence makes the α-factor particularly disposed to binding to copper(II) ions [88]. The copper(II)/α-factor complex itself is prone to forming a ternary complex with other ligands containing an imidazole group or proteins containing surface histidine residues.

A question remains to be addressed: what caused the change to the dielectric properties of the plasma membrane upon addition of α-factor? One potential explanation would be that the binding of the α-factor induced a change in width of the membrane in the vicinity of bound Ste2. A simple width alteration to the plasma membrane layer, or shell, would change the specific capacitance, $C_{cm}=\frac{\varepsilon_{o}\varepsilon_{cm}}{d_{cm}}$, and, as a by-product of assuming a constant $d_{cm}$ for both treated and untreated samples in the three-shell model used to simulate the dielectric measurements, instead be reflected as a change in $\varepsilon_{cm}$. However, this change in width of the membrane would not simply be due to the physical presence of the additional layer of α-factor molecules surrounding the cell. It is well-accepted that the contribution to the measured dielectric spectra arising from the region of the plasma membrane in the MHz frequency range is dominated by the hydrophobic core of the membrane [49,89,90], so simply stacking ligand molecules to the outer recesses of the plasma membrane layer should not have a large effect on the membrane dielectric properties. Furthermore, if the α-factor layer caused an increase in the width of the plasma membrane from the perspective of the dielectric measurements, the capacitance of the untreated cells would actually be higher than the Ste2-expressing cells that were bound to ligand (which presumably would have an increased width due to the layer of α-factor molecules). However, the measured value of the plasma membrane permittivity was lower for the untreated cells in our study. 

Any change to the width of the plasma membrane caused by binding of α-factor and sensed by the dielectric measurements would more likely be due to a deformation of the membrane caused by a change to the conformation of the GPCR, and specifically, a change to the hydrophobic length of one or more of the TM domains of the GPCR. Molecular dynamics simulations have shown that the plasma membrane in the near vicinity of a membrane embedded receptor can deform, i.e., increase or decrease its hydrophobic thickness, to alleviate the mismatch between the protein’s hydrophobic length and the membrane’s hydrophobic thickness [91]. Binding of a ligand can alter the conformational state of the receptor and hence alter the level of hydrophobic mismatch between the receptor and surrounding membrane, which would in turn produce a deformation of the nearby membrane. Therefore, changes to the conformation of a receptor which result in a reduced hydrophobic exposure of one or more of the TM domains would result in a decrease in thickness of the membrane in the regions adjacent to said domain to account for the mismatch. In fact, Shan et al. have shown, via MD simulation, that ligand-induced conformational rearrangements produced by three different ligands to the Serotonin 2A receptor provoked three different rearrangements in the thickness of the lipid membrane surrounding the receptor [92]. It is also feasible that the changes to the plasma membrane permittivity sensed in the dielectric measurements are due to a rearrangement of the receptor locations within the membrane caused by agonist binding. Simulation studies have shown that for certain receptor agonist systems, membrane receptors exposed to saturating concentrations of agonist undergo migration into more densely packed lipid regions of the membrane [93]. While, far from proving such simulations predicting lipid raft-mediated receptor migration or changes in curvature of the membrane as a result of GPCR conformational changes, the results presented in this manuscript demonstrate the potential of dielectric spectroscopy in monitoring small but significant changes due to cellular processes such as those suggested above. In order to detect the presence of alpha factor at the plasma membrane, we focused on the structural Maxwell–Wagner relaxation, which occurs below 110 MHz. Future studies could expand the frequency range probed by the dielectric measurements and explore whether the ligand could be detected using microwaves as well.

The fact that dielectric spectroscopy proved to be sensitive enough to detect changes in dielectric properties associated with various subcellular regions induced by treatment with ligand is very encouraging; however, due to the large number of morphological and electrical parameters needed to properly simulate the dielectric data, two populations of cells must be prepared as close to identical as possible in order to properly compare them. Hence, experiments in which the same exact cells can be measured before and after addition of ligand are perfect for the utilization of this technique. In this regard, we feel the combination of this study with technology allowing for single cell dielectric measurements [94], and in particular devices that allow for the cell suspending medium, e.g., microfluidic devices incorporating measurement electrodes [34,95,96] to be exchanged between measurements, should catapult dielectric spectroscopy to the forefront of relevance with regard to the intricate relationships between plasma membrane receptors, binding ligands, and lipids.

## Figures and Tables

**Table 1:** Average difference between electrical phase parameters, plus or minus one standard deviation (n = 6 experiments), along with the *p*-value obtained by applying a paired Student’s *t*-test to pairs of Ste2-expressing cell suspensions, one with and one without α-factor added to it.

	$\varepsilon_{w}$	$\kappa_{w}(\mathrm{mS}/m)$	$\varepsilon_{cm}$	$\varepsilon_{cp}$	$\kappa_{cp}\;(S/m)$
$\langle \delta_{m}\rangle$	−0.13 ± 2.4	0.0 ± 0.0	0.06 ± 0.05	−11.4 ± 9.2	−0.035 ± 0.027
*p*	0.90	1.0	0.026	0.029	0.025

**Table 2:** Average difference between electrical phase parameters, plus or minus one standard deviation (n = 5 experiments), along with the *p*-value obtained by applying a paired Student’s *t*-test to pairs of Ste2Δ cell suspensions, one with and one without α-factor added to it.

	$\varepsilon_{w}$	$\kappa_{w}\;(\mathrm{mS}/m)$	$\varepsilon_{cm}$	$\varepsilon_{cp}$	$\kappa_{cp}\;(S/m)$
$\langle \delta_{m}\rangle$	−2.4 ± 3.5	0.0 ± 0.0	0.00 ± 0.04	−3.5 ± 2.1	−0.015 ± 0.006
*p*	0.21	1.0	0.84	0.020	0.007

**Table 3:** Average difference between electrical phase parameters, plus or minus one standard deviation, (n = 4 experiments) along with the *p*-value obtained by applying a paired Student’s *t*-test to pairs of Ste2Δ cell suspensions suspended in electrolytic solution with and without Sorbitol added to it.

	$\varepsilon_{w}$	$\kappa_{w}\;(\mathrm{mS}/m)$	$\varepsilon_{cm}$	$\varepsilon_{cp}$	$\kappa_{cp}\;(S/m)$
$\langle \delta_{m}\rangle$	0.1 ± 3.8	1.25 ± 2.5	0.00 ± 0.07	−0.2 ± 14.3	−0.002 ± 0.04
*p*	0.96	0.39	1.0	0.98	0.94

**Table 4:** Average difference in cellular and vacuolar radii plus or minus one standard deviation (n = 3 experiments) along with the *p*-value obtained by applying a paired Student’s *t*-test to pairs of for Ste2-expressing and Ste2Δ cells suspended in electrolytic solution with and without α-factor added to them.

	Ste2-Expressing Cells		Wild Type Cells	
	*R* (µm)	*R_o_* (µm)	*R* (µm)	*R_o_* (µm)
$\langle \delta_{m}\rangle$	−0.01 ± 0.05	0.00 ± 0.08	−0.05 ± 0.06	−0.02 ± 0.02
*p*	0.85	0.95	0.29	0.18

## Data Availability

The data presented in this study are openly available in the FigShare Repository at 10.6084/m9.figshare.14502006.
